# Melatonin as a Potential Multitherapeutic Agent

**DOI:** 10.3390/jpm11040274

**Published:** 2021-04-06

**Authors:** Yulia Baburina, Alexey Lomovsky, Olga Krestinina

**Affiliations:** Institute of Theoretical and Experimental Biophysics, Russian Academy of Sciences, Pushchino, 142290 Moscow, Russia; byul@rambler.ru (Y.B.); lomovskyalex@gmail.com (A.L.)

**Keywords:** melatonin, mitochondrial dysfunction, oxidative stress, aging, heart failure, cancer

## Abstract

Melatonin (N-acetyl-5-methoxytryptamine, MEL) is a hormone produced by the pineal gland that was discovered many years ago. The physiological roles of this hormone in the body are varied. The beneficial effects of MEL administration may be related to its influence on mitochondrial physiology. Mitochondrial dysfunction is considered an important factor in various physiological and pathological processes, such as the development of neurodegenerative and cardiovascular diseases, diabetes, various forms of liver disease, skeletal muscle disorders, and aging. Mitochondrial dysfunction induces an increase in the permeability of the inner membrane, which leads to the formation of a permeability transition pore (mPTP) in the mitochondria. The long-term administration of MEL has been shown to improve the functional state of mitochondria and inhibit the opening of the mPTP during aging. It is known that MEL is able to suppress the initiation, progression, angiogenesis, and metastasis of cancer as well as the sensitization of malignant cells to conventional chemotherapy and radiation therapy. This review summarizes the studies carried out by our group on the combined effect of MEL with chemotherapeutic agents (retinoic acid, cytarabine, and navitoclax) on the HL-60 cells used as a model of acute promyelocytic leukemia. Data on the effects of MEL on oxidative stress, aging, and heart failure are also reported.

## 1. Introduction

Melatonin ((N-acetyl-5-methoxytryptamine, MEL) is an indoleamine that is produced by the pineal gland and other organs such as the skin, bone marrow, retina, and gastrointestinal tract [1,2]. MEL is a derivative of the biogenic amine serotonin, which, in turn, is synthesized from the amino acid tryptophan. After synthesis in the epiphysis, MEL enters the cerebrospinal fluid and blood. The concentration of the hormone produced in the pineal gland depends on the time of day; about 70% of all the MEL in the body is produced at night. The synthesis of MEL in the body depends on light: under conditions of excessive (daytime) light, the synthesis of the hormone decreases, and when there is reduced light, it increases [3]. Since MEL is a lipophilic molecule, it easily penetrates the cell membranes to reach subcellular compartments [4]. It was found that MEL is not only produced by the pineal gland. The amount of MEL produced in the brain would not be enough to maintain vital processes and regulate the rhythm of sleep and wakefulness. Therefore, two components of the MEL production system are considered: central, through the pineal gland, where the synthesis of the sleep hormone depends on changes in light and darkness, and peripheral, involving the rest of the cells in which the production of MEL is not associated with light. These are cells that are common throughout the human body, including cells of the walls of the gastrointestinal tract, cells of the lungs and respiratory tract, cells of the renal cortex, blood cells, etc. [3,5,6].

It is generally accepted that the main function of the hormone MEL is to regulate the body’s circadian rhythm [5]. However, upon further and careful study of MEL and its effects on the human body, scientists have established that this substance has other important and beneficial properties for humans. MEL ensures the effective functioning of the endocrine system of the body, slows down the aging process in the body, and helps the body to adapt to changes in the time zone. Moreover, MEL stimulates the protective functions of the body’s immune system, has an antioxidant effect, helps the body fight stress, and is associated with the manifestation of seasonal depression [6]. Additionally, MEL regulates the cardiovascular system and blood pressure, participates in the work of the body’s digestive system, affects the production of other hormones in the body, and has a positive effect on human brain cells [7].

As mentioned above, MEL exhibits anti-inflammatory, anticancer, and antioxidant effects and has both pro-apoptotic and anti-apoptotic properties. The opposing actions of MEL are associated with the receptor-dependent and receptor-independent mechanisms of this indolamine [8,9,10].

Most of the beneficial effects of MEL administration may depend on its effect on mitochondrial physiology [11,12]. Mitochondrial dysfunction is considered an important factor in a variety of physiological and pathological situations, such as neurodegenerative and cardiovascular diseases, diabetes, various forms of liver disease, skeletal muscle disorders, and aging [13,14,15]. Changes in mitochondrial function, in particular, changes in the activity of the electron transport chain and oxidative phosphorylation have been proposed as the main causative factors in the pathogenesis of these disorders. Numerous studies have shown that MEL plays an effective role in maintaining mitochondrial homeostasis [14,16,17]. This may explain the protective effect of this molecule in several physio-pathological conditions, including neurological conditions [16,18,19], cardiovascular diseases [17,20,21], and aging [22], all of which are associated with mitochondrial dysfunction.

It was shown that the actions of MEL in the mitochondria can be directed to the regulation of the Ca^2+^-induced cyclosporin A-sensitive pore or mitochondrial permeability transition pore (mPTP), which is formed due to the accumulation of suprathreshold Ca^2+^ concentrations or in response to oxidative stress and is considered the initial stage of apoptosis [23]. Jou and coauthors found that the introduction of MEL is able to not only prevent the induction of mPTP but also maintains the mitochondrial potential (ΔΨm) and maintains ΔΨm-dependent formation of ATP [24]. It should be noted that the effect of MEL on mitochondria can vary depending on the experimental conditions. MEL also has a high level of specificity in relation to different organs and tissues [25]. Along with the antiapoptotic effect of MEL, it has been shown that MEL induces mitochondrion-mediated apoptosis in HL-60 cells [26]. Moreover, MEL can act as a reducing or oxidizing agent depending on the structural features of the target molecules and their environment, the incubation time, and the concentrations used [27].

In this review, we present our findings, which shed light on the multiple functions of MEL in pathologies such as aging, cancer, and heart failure.

## 2. Melatonin and mPTP

The free distribution and functional diversity of MEL determine a wide range of its functions. At the moment, in addition to the regulation of circadian rhythms, anti-inflammatory, antioxidant, and oncostatic actions have been shown [28]. There is also a significant amount of data on the effect of MEL on mitochondrial function [11,12,29,30,31,32,33]. It is well known that the mitochondria play key roles in a large number of physiological processes in the cells, and mitochondrial damage is considered the main cause of many pathological processes, including aging, ischemia/reperfusion injury, septic shock, and neurodegenerative diseases [34,35]. Due to its small size and amphiphilic nature, MEL can reach numerous cellular and subcellular structures, in particular, the mitochondria, and accumulate in the mitochondria in concentrations hundreds of times higher than those in the cytosol, which increases the possibility of its functional significance in mitochondrial bioenergetic processes [36,37]. Numerous studies have shown that MEL is able to improve the functional state of the mitochondria; for example, the chronic administration of MEL at pharmacological doses prevents mitochondrial dysfunction in models of experimental diabetes and intoxication, showing the mitochondrion-specific effect of MEL [38,39,40]. MEL is able to exhibit antioxidant properties by binding free radicals of all types [41], directly reducing the toxic effects of reactive oxygen species (ROS), as well as restoring the activity of antioxidant enzymes and preventing an increase in mitochondrial membrane permeability [42]. Some time ago, investigators showed that the target of MEL action in the mitochondria could be the mPTP, which is formed due to the accumulation of threshold concentrations of Ca^2+^ or in response to oxidative stress and is considered the initial stage of apoptosis [29,31,42]. Ca^2+^-induced pore opening leads to depolarization of the inner membrane, dissociation of respiration and phosphorylation, as well as swelling of the mitochondria and the release of proapoptotic factors [43]. A number of molecules have been accepted as being key regulators of the mPTP, including Cyclophilin D (Cyp-D), in the matrix [44], Adenine nucleotide translocase (ANT) and phosphate carriers in the inner mitochondrial membrane, and voltage-dependent anion channels (VDACs) in the outer mitochondrial membrane [43,45]. Moreover, dimers of F_o_F_1_-ATP synthase have been suggested to be new putative regulators of the mPTP [46,47]. MEL, due to its structure, has many different functions and, as a consequence, many mechanisms of action on the mPTP. Different pathways of MEL action on the mPTP can be manifested depending on the structural features of the target molecules and their environments, incubation time, and used concentrations. There are many works describing the inhibitory effect of MEL on the opening of the mPTP and, therefore, its anti-apoptotic effect in normal and various pathologies [24,29,30,31,32,42,47,48,49]. For example, Jou and coauthors demonstrated the formation of the mPTP in the mitochondria of astrocytes [24]. They showed that MEL does not directly inhibit the mPTP but is able to prevent a drop in the membrane potential and depolarization and retain ATP formation during disturbed Ca^2+^ homeostasis [24]. Direct inhibition of the mPTP by MEL has also been reported [50]. The effect of MEL on mPTP currents at the single channel level was studied using the patch-clamps method in rat liver mitoplasts. It was shown that MEL directly inhibits the mPTP in a dose-dependent manner [50]. Studies by Petrosilio’s group linked the inhibitory effect of MEL to the discovery of mPTP with cardiolipin peroxidation [51]. Cardiolipin on the outer surface of the inner mitochondrial membrane binds to cytochrome *c*, and when cardiolipin is oxidized, cytochrome *c* molecules are detached and released from the mitochondria [52]. The influence of cardiolipin on the induction of the opening of the mPTP has been shown [53], suggesting that it can play a coordinated role in this process by interacting with the components of the mPTP, probably via the carrier of adenine nucleotide and/or with the dimers of ATP synthase [51]. Moreover, it has been shown that micromolar concentrations of MEL can suppress cardiolipin peroxidation while also inhibiting the mPTP [53]. The authors also showed that stress-induced release of cytochrome *c* from the mitochondria is completely prevented by MEL, which can be explained by the ability of MEL to prevent cardiolipin peroxidation, inhibiting the deallocation of cytochrome *c* from the inner mitochondrial membrane [54]. Inhibitory effects of MEL on the mPTP and the 5-hydroxydecanoate-induced KATP channels in isolated brain mitochondria have also been found [48]. The authors showed that the inhibitory effect of MEL in the rat brain mitochondria is associated with the actions of another selective inhibitor of the mPTP, cyclosporin A (CsA), which makes it possible to use MEL, and drugs based on it, in the development of therapeutic strategies for the treatment of neurodegenerative diseases [48].

Our own studies suggest an important role for MEL in the development of mitochondrial dysfunction during aging and, in addition, an anti-apoptotic protective effect when administered chronically [55]. MEL improved the functioning of the brain mitochondria, prevented the appearance of the mPTP on the inner membrane and, therefore, improved energy supply to the brain. Chronic MEL administration has been shown to be capable of maintaining the stability of the inner membrane which, in turn, may be important for optimal electron transport and energy transduction [29].

Moreover, according to our data, MEL inhibited mPTP and, thus, exhibited a protective effect on the mitochondria of the rat heart under conditions of acute heart failure [32]. We observed the effect of MEL administration on mPTP functioning in heart mitochondria from aged rats with acute cardiac failure caused by isoprenaline hydrochloride (ISO). In our experiments, ISO induced the opening of the mPTP in the rat heart mitochondria (RHM), decreasing the Ca^2+^ retention capacity, while MEL prevented the ISO effect and increased the Ca^2+^ retention capacity [32]. Thus, the protective antiapoptotic properties of MEL in various diseases reveal its possible potential to be used as a therapeutic agent in the treatment of diseases with various etiologies.

Nevertheless, there is growing evidence of the contradictory actions of MEL in the mitochondria and mPTP depending on the conditions. Martinis and coauthors showed that the presence of MEL in isolated rat liver mitochondria caused the induction of the mPTP, an increase in oxidative stress, and the release of proapoptotic factors [25]. These effects were only manifested in the presence of threshold concentrations of Ca^2+^ and were inhibited by the action of CsA, which demonstrates the relationship of this phenomenon with the mPTP. The authors suggested that this pro-apoptotic effect of MEL is associated with oxidative stress caused by threshold concentrations of Ca^2+^ and MEL, which leads to the formation of hydrogen peroxide, and, as a consequence, to the oxidation of glutathione, thiol groups, and pyridine nucleotides [25]. Our results also show that direct addition of MEL to rat brain mitochondria leads to initiation of the mPTP and the induction of apoptosis [10]. Moreover, under these conditions, the expression of 2′,3′-cyclic nucleotide 3′-phosphodiesterase (CNPase) and VDACs, as regulators of mPTP, was decreased. We explain this effect of MEL by the existence of a complex of proteins in mitochondrial membranes, including CNPase and VDACs, which is able to take part in the regulation of the mPTP and may be the target of the direct action of MEL in the mitochondria [10]. We have previously shown that decreases in CNPase and VDAC expression lead to the initiation of the mPTP [56]. CNPase and VDAC are colocalized [57]. In addition, CNPase is associated with complexes of the respiratory chain [57]. The addition of MEL to mitochondria induces the opening of the mPTP, which can lead to CNPase release from the mitochondria [57] and rupture of the relationship with VDACs. Since 2′,3′-AMP is a substrate of CNPase, as a result of a decrease in the CNPase content, an increase in free 2′,3′-AMP can occur. The accumulation of harmful 2′,3′-AMP may result in the initiation of the mPTP and, as a result, cell death. In addition, some time ago, it was shown that the treatment of HL-60 cells with MEL led to the depolarization of mitochondrial membranes and induction of the mPTP. In addition, the effects of MEL on cells resulted in the activation and association of the proapoptotic proteins Bax and Bid and also contributed to detectable increases in the expression of both proteins [26]. Later, we observed the ability of MEL to enhance the proapoptotic effects of some chemotherapeutic agents, such as retinoic acid (ATRA), Navitoclax (ABT-737), and cytarabine (CYT), in HL-60 cells [58,59,60]. We showed that MEL has the ability to not only suppress the expression of antiapoptotic proteins of the Bcl family (Bcl-2 and Bcl-xL) but also to regulate the expression of proteins considered to be the most important regulators of the mPTP (VDAC, TSPO, and CNPase) [59]. This gives us reason to believe that the proapoptotic effect of MEL under these conditions is associated with the functioning of the mPTP, and we suggest that MEL could be used as a potential proapoptotic and oncostatic agent. In general, it should be noted that the mechanism of action of MEL on the mPTP and, therefore, on mitochondrion-mediated apoptosis, remains unclear. There appears to be several pathways of MEL exposure, depending on the conditions and environment, allowing MEL to act as both an inducer and an inhibitor of the mPTP. This makes it possible to reveal the increased potential of MEL and to develop drugs based on this as a strategy for the treatment of various diseases.

## 3. The Role of Melatonin in Pathologies

Currently, MEL and drugs based on it are used in medicine, mainly for the treatment of disorders associated with the regulation of circadian rhythms of sleep and wakefulness. However, due to the wide range and variety of properties it manifests, MEL has the potential to be used for various diseases with various etiologies. Thus, there is a large amount of data on the oncoprotective properties of MEL [61,62].

There is also evidence of a positive effect of MEL in alcoholic intoxication. For example, MEL protects against alcoholic liver injury by attenuating oxidative stress, the inflammatory response, and apoptosis in Kupffer cells of mice [63]. It has also been shown that MEL is able to reduce pathological damage to the liver caused by the gastric perfusion of alcohol. Authors have stated that MEL exerts protective effects against alcohol fatty liver disease induced by alcohol in rats probably through two modes of action: antioxidant actions and actions mediated by its receptors [64]. Data from some laboratories indicate the ability of MEL to eliminate damage to the lungs [65] and blood vessels [66] caused by smoking.

Several studies have been devoted to the effect of melatonin on infections caused by Helicobacter pylori [67,68,69]. The addition of melatonin to the standard H. pylori treatment protocol has been shown to increase its effectiveness, accelerate healing, and help to relieve pain syndromes [69]. A group of scientists from China demonstrated the effectiveness of melatonin in preventing gastrointestinal motility disorder and stress-induced gastric ulcers [70]. The authors attribute this effect to its ability to reduce oxidative stress and regulate gut hormones [70]. In addition, melatonin can be used as an agent for the treatment of the pancreas and kidneys, as it has been shown to be effective for treating acute pancreatitis [71], ulcerative and lymphocytic colitis [72], and other kidney diseases [73].

Finally, last year, under the direction of Professor Reuter, authors published several articles claiming that MEL could effectively treat the new COVID-19 infection [74,75,76,77,78]. Moreover, the authors suggested that MEL may be useful in the fight against COVID-19-induced disorders of various organs e.g., heart [74,76] and lungs. These assumptions certainly need confirmation and expand the possibilities of further use of MEL in medicine and pharmacology.

### 3.1. Melatonin in Aging

Earlier in this review, the multiple effects of MEL on mitochondria were discussed. Undoubtedly, MEL strongly influences the functioning of the mitochondria, regulates their activity, and protects them against possible damage due to pathologies. It is widely known that mitochondrial dysfunction underlies the mechanisms of development of many diseases of various etiologies, including liver diseases, cardiovascular diseases, diabetes, as well as neurodegenerative and muscle disorders. Processes associated with aging are also directly related to mitochondrial dysfunction. A decrease in the activity of the Ca^2+^-transporting system, oxidative stress, and an increase in the production of ROS and in the permeability of the inner mitochondrial membrane, which occurs due to suprathreshold concentrations of Ca^2+^ in the matrix, are considered key events in age-related dysfunctions of mitochondria [79,80,81,82]. MEL, being a strong natural antioxidant, is able to reduce age-related oxidative damage. Many researchers associate the protective effect of MEL on the mitochondria during aging primarily with its scavenging potential in the mitochondrial matrix and in the intermembrane space [83,84,85]. Thus, MEL is able to increase the activity of complexes II, III, and IV of the mitochondrial respiratory chain while reducing the amount of ROS in aged rats [86].

In addition, aging leads to an increase in mitochondrial sensitivity to the opening of the mPTP, which leads to depolarization of the inner membrane, disconnection of respiration and phosphorylation, as well as swelling of the mitochondria and the release of proapoptotic factors [87,88]. Our research and data from other laboratories show the ability of MEL to restore the function of the mPTP in aged animals [29,31,55,84,89]. There appear to be several mechanisms of MEL action on the mPTP. Studies by Paradise’s group showed that the effect of MEL on the pores are associated with its ability to prevent cardiolipin oxidation [84]. The oxidation of cardiolipin is associated with numerous types of mitochondrial dysfunction, including the effects of aging [90,91]. The oxidized form of cardiolipin, when added to mitochondria, together with threshold Ca^2+^ concentrations, promotes the opening of the mPTP [41]. This cooperative action can probably be explained by the conformational transitions of the components of the mPTP complex, facilitating its opening [84]. Moreover, ROS production mediated by the respiratory chain, induces cardiolipin peroxidation, which, in turn, facilitates the detachment of cytochrome *c* from the inner mitochondrial membrane, the opening of the mPTP, and the release of cytochrome *c* from the mitochondria. Melatonin prevents this cascade of events by inhibiting cardiolipin peroxidation [54].

In previous studies conducted in our laboratory, we demonstrated that long-term treatment of old rats with MEL increases the Ca^2+^ capacity, slows down mitochondrial swelling, and prevents the release of proapoptotic factors (such as Cyt c) from the rat brain and liver mitochondria during aging, thus improving the mitochondrial functional state [89]. In addition, according to our data, MEL is able to prevent stress-induced opening of the mPTP in the non-synaptic brain mitochondria of old rats [55]. We induced oxidative stress by adding cumene hydroperoxide to the mitochondria and showed that in old rats fed with MEL for a long period of time, the mitochondria were protected from oxidative stress at both low (1 μM) and high (100 μM) oxidant concentrations. At the same time, in young animals, MEL only prevented minor oxidative stress caused by a low concentration of the oxidant (1 μM) [55]. These data indicate a greater sensitivity of age-related mitochondria to treatment with MEL, which indicates the potential to use it for the treatment of age-related patients with various diseases.

The results of our more recent studies have led us to suggest that one of the targets of MEL action in the mitochondria is 2′,3′-cyclic nucleotide 3′-phosphodiesterase (CNPase), an enzyme that is the main integral protein of myelin but also has extensive actions in unmyelinated tissues [89]. CNPase hydrolyzes 2′,3′-cyclic nucleotides to the corresponding monophosphates [92]. In the mitochondria, CNPase is involved in the regulation of the mPTP, and when the pore is opened, it can be released from the mitochondria in parallel with the release of proapoptotic factors [57]. In addition, we showed that an increase in the sensitivity of mitochondria to the opening of the mPTP during age-related changes is associated with a decrease in the amount of mitochondrial CNPase [93]. A decrease in the amount of enzyme activity of CNPase in the mitochondria with age leads to an increase in 2′,3′-cAMP [94] and, at the same time, the intracellular accumulation of 2′, 3′-cAMP acts as a pro-apoptotic signal through the functioning of the mPTP [56,93]. However, in the mitochondria of the brains and livers of old rats subjected to long-term treatment with MEL, a previous study showed that the sensitivity to the discovery of the mPTP decreased, the rate of mitochondrial swelling decreased, and the Ca^2+^-induced release of cytochrome *c* and CNPase were also prevented compared with a control treatment [89]. Additionally, the mitochondria of old rats have the ability to accumulate orally administered MEL to a greater extent than the mitochondria of young rats, while the level of enzyme activity of CNPase in such rats is also higher than in control animals (not treated with MEL) [89]. Thus, chronic administration of MEL in old animals contributes to the retention of CNPase in the mitochondria, prevents the opening of mPTP, and thus improves mitochondrial function and decreases mitochondrial sensitivity to stress [89].

### 3.2. Melatonin and Cancer

Currently, cancer is one of the main problems for humanity. Despite many years of research in this area, the study of the problem of the appearance and development of malignant tumors is extremely relevant. Therefore, it is necessary to analyze new drugs and/or combinations of already known drugs designed to induce cell death in order to increase the effectiveness of antitumor therapy.

Many studies have proved the antitumor effect of MEL in various types of tumor cells. Abnormal levels of MEL in cancer patients indicate that it can be considered to be a physiological oncostatic substance [61,62]. Moreover, it was found that MEL inhibits the development of cancerous tumors at the stages of initiation, progression, and metastasis [95]. However, combined therapy with MEL and chemotherapy drugs is poorly understood. In our work, we investigated the combined effect of MEL and chemotherapeutic agents from different pharmacological groups—retinoic acid (ATRA), cytarabine (CYT), and navitoclax (ABT-737)—on the proliferation, differentiation, and expression of mitochondrial target proteins in HL-60 cells used as a model of acute promyelocytic leukemia (APL) [58,59,60]. It is known that ATRA is used clinically for the treatment of APL as a differentiating agent in combination with chemotherapeutic drugs [96,97].

Combination therapy with retinoic acid (ATRA) and chemotherapy drugs was shown to significantly increase remission and disease-free survival [98,99]. The detrimental effects of ATRA (for example, ATRA syndrome) and the toxicity of chemotherapeutic agents continue to be relevant challenges in the treatment of APL [100,101]. MEL has beneficial effects on a variety of cancers, including pancreatic, liver, and prostate cancer cells [102,103,104]. MEL is known to induce apoptosis in HL-60 cells [26,105]. In addition, MEL increases the effectiveness of other chemotherapy drugs [106]. We showed the effect of MEL in combination with ATRA, CYT, and ABT-737 at concentrations lower than those used in medical practice (10, 2, and 0.2 μM, respectively) on the activation of HL-60 cell proliferation. MEL reduced cell viability; however, combined treatment of MEL (1 mM) with ATRA (10 nM), MEL (1 mM) with CYT (2 nM), and MEL (1 mM) with ABT-737 (0.2 μM) at non-toxic concentrations increased cytotoxicity towards HL-60 cells compared to treatment with MEL, ATRA (10 nM), CYT (2 nM), and ABT-737 (0.2 μM) applied separately [58,59,60].

Moreover, the combined use of MEL with ATRA, MEL with CYT, and MEL with ABT-737 significantly suppressed the expression of the antiapoptotic protein Bcl-2 compared to protein expression after treatment with ATRA (10 nM), CYT (2 nM), ABT-737 (0.2 μM), or MEL. It is possible that the decrease in the Bcl-2 level under the effect of MEL with ATRA, CYT, or ABT-737 triggers the apoptosis signaling cascade. The initial stage of apoptosis involves the formation of the mPTP in the inner mitochondrial membrane, which is accompanied by a drop in membrane potential, swelling of the mitochondria, and the release of cytochrome *c* from the intermembrane space into the cytosol through the permeability of the outer mitochondrial membrane [107]. Treatment with MEL in combination with ABT-737 (0.2 μM) reduced the Ca^2+^ capacity, increased the decrease in membrane potential while increasing the production of ROS, and suppressed the expression of antiapoptotic proteins, such as Bcl_xL_, Bcl_w_, and Mcl-1, but increased the expression of proapoptotic BAX. In addition, MEL in combination with ABT-737 (0.2 μM) caused depolarization of the mitochondrial membrane, increasing the decrease in membrane potential, which could lead to an increase in ROS production with a subsequent increase in autophagy [59].

It is known that the mitochondria of cancer cells are structurally and functionally different from normal mitochondria [108,109]. In addition, tumor cells undergo extensive metabolic reprogramming, which makes them more susceptible to mitochondrial damage than nonimmortalized cells [110,111]. On this basis, agents targeting mitochondria can be considered agents for selectively targeting tumors.

The correction of mitochondrial dysfunction associated with cancer and reactivation of cell death programs with pharmacological agents affecting mitochondrial membrane permeabilization are attractive strategies for cancer treatment [112,113,114].

Mitochondrial VDACs are a major component that can target tumors. VDACs initiate apoptotic-signaling cascades and, therefore, are able to deplete the metabolic flux of tumors. Molecular interactions of VDACs with pro- and antiapoptotic proteins of the outer mitochondrial membrane are multifaceted and can both promote and prevent cell death [110,111,112,113,114,115]. We found that VDAC1 expression in HL-60 cells decreased after combined use of ATRA (10 nM) and MEL or MEL and CIT, indicating changes in the regulation of metabolic and energetic functions of the mitochondria of cancer cells. The partner and modulator of VDACs is a translocator protein (TSPO) located in the outer membrane of the mitochondria [116]. TSPO has been found to be involved in the regulation of cell proliferation and apoptosis in gliomas, which are highly aggressive malignant tumors with a poor prognosis [117]. TSPO colocalizes with VDACs and forms a strong complex with these proteins. It can modulate VDAC conductance and regulate mitochondrial function under various physiological and pathophysiological conditions [118].

Many researchers have reported that TSPO expression is increased in various types of cancer, including brain tumors and gliomas. [119,120,121]. In addition, increased TSPO expression is correlated with oncogenicity [122]. We observed a decrease in TSPO expression in HL-60 cells treated with ATRA (1 μM), MEL and ATRA (10 nM), and MEL and CYT. Presumably, a decrease in TSPO expression under these conditions can cause a decrease in tumorigenicity [58,60,122].

Acuna-Castroviejo and colleagues showed that MEL is able to improve the activity of mitochondrial respiratory chain complexes, reducing the formation of ROS at the complex I and IV levels [123]. High mitochondrial complex activity is necessary for the normal functioning of cells. Therefore, in our investigation, we analyzed changes in the levels of the main subunits of the electron transport chain (ETC) complexes under the effect of MEL in combination with ATRA in HL-60 cells [58]. The activity of ETC complexes in HL-60 cells must be reduced to prevent tumor development. The combined effect of ATRA (10 nM) with MEL reduced the expression of subunits of the ETC complexes and, therefore, their activity.

All of these data indicate the important role of the pineal gland in the development of cancer. The suppression of its function under constant illumination stimulates carcinogenesis. The use of the epiphyseal hormone MEL inhibits carcinogenesis in both normal light conditions and under constant illumination. This means that MEL can be very effective for the prevention of cancer, especially in the northern regions, where it is always light in summer (“white nights”), and during the long polar nights, electric light burns everywhere. All of the above information indicates the potential of MEL to act as a pro-apoptotic agent, which may have important biological, therapeutic, and pharmacological significance.

### 3.3. Melatonin and Heart Failure

Mitochondria play a key role in the normal functioning of the heart as well as in the pathogenesis and development of various types of heart disease. Physiologically, mitochondrial ATP production is consistent with heart ATP consumption, which is largely controlled by mitochondrial Ca^2+^ transport pathways that regulate [Ca^2+^] levels in mitochondria [124]. An increase in the concentration of Ca^2+^ in the mitochondrial matrix activates dehydrogenase enzymes that increase the level of NADH and, consequently, accelerate the production of ATP. In pathology, mitochondrial Ca^2+^ is important for the generation of ROS as well as for the opening of the mPTP, factors involved in both the occurrence of post-ischemic heart damage and the development of heart failure [124].

Heart failure is a highly common condition with a poor prognosis. Despite the fact that effective treatment methods have been developed over the past 20 years, mortality from heart failure remains quite high and the search for new therapeutic approaches for heart failure is urgent [125]. Much attention is being paid to research aimed at enhancing the protective response to oxidative stress with various antioxidants to reduce age-related oxidative damage and mitochondrial dysfunction [126]. Antioxidants play the role of the defense system in organisms. Among the effective antioxidants, MEL should be highlighted, since it is a natural compound—a neuroendocrine hormone produced by the pineal gland. MEL is a well-known antioxidant that effectively protects mitochondrial bioenergetic functions. MEL is a lipophilic molecule that penetrates cell membranes, easily reaching subcellular structures [4]. As we age, the level of MEL decreases, and therefore its protective properties are weakened. In cells, MEL is found in the nuclei and mitochondria, and in the latter, it accumulates in high concentrations [126]. Researchers from different laboratories showed that chronic treatment with MEL in pharmacological doses affects mitochondrial functions and prevents their disturbances in an experimental model of diabetes and after intoxication, demonstrating the specific activity of MEL in mitochondria [127,128,129].

There is evidence in the literature that the effect of MEL is associated with a decrease in blood pressure in an experimental model of hypertension and in a clinical setting [130] under limitations of myocardial infarction [131] and post-infarction remodeling, and it has antifibrotic effects on hypertensive hearts [132]. However, the data on the effects of MEL in heart failure are poorly understood.

We conducted a study of the effect of MEL on the functional state of cardiac mitochondria isolated from aged rats with acute heart failure induced by isoprenaline hydrochloride (ISO) injection upon opening of the mPTP [32].

To study the effect of MEL on the heart mitochondria in acute heart failure, we used a model of chronic MEL administration according to the Petrosillo method [133], which is recognized in the scientific community and was modified in our laboratory [32]. Acute heart failure in rats was induced by intraperitoneal administration of ISO [134].

Histological analysis of cryosections of the left ventricle (LV) of rat hearts showed that the control samples had standard parameters of the structure and architectonics of the myocardium of this age group of Wistar rats. LV tissue samples from rats treated with MEL differed markedly from controls; the structure of muscle fibers was better preserved, and age-related changes were less pronounced. In LV tissue samples from rats injected with ISO, significant myocardial degeneration was observed, and this was manifested by muscle fiber swelling, complete blurring of the contours of myofibrillar cells, and vacuolization of the sarcoplasma, granular degeneration, the appearance of myolysis foci with lumpy fiber disintegration, and the development of small focal necrosis with micromalacia (up to the disappearance) of cell nuclei. At the same time, in LV tissue samples from rats injected with MEL and injected with ISO, partial degenerative changes were also observed in the myocardium, and these manifested in fine-focal homogenization of myofibrils with smoothing (up to complete disappearance) of the transverse hatching and a loss of intramyofibrillar contours as well as the appearance of localized vacuolization sarcoplasm without pronounced swelling of muscle fibers. The absence of the pronounced swelling of muscle fibers and the demarcation localization of the affected area caused by the introduction of ISO indicates the protective effect of MEL taken by experimental animals.

Next, we investigated the functional parameters of mitochondria isolated from the four studied groups of rats. We have shown that an important characteristic of mitochondrial integrity, the value of respiratory control, decreased in rats injected with ISO, while chronic consumption of MEL, which preceded the introduction of ISO, contributing to the prevention of this decrease, while chronic administration of MEL did not affect the respiratory control of mitochondria isolated from 12-month-old rats.

In the next step, we examined the effects of MEL and ISO on mPTP function in the mitochondria of rat hearts. We were showed quantitative changes in the Ca^2+^-capacity of mitochondria isolated from each experimental group of rats. We noticed that MEL increased the Ca^2+^ capacity by 55%, while ISO decreased it by 40%. However, when ISO was administered to rats that consumed MEL, the ability of the mitochondria to retain Ca^2+^ did not change compared with the control (RHM from group 1), while compared with RHM from group 3, it increased by 47%. Thus, we concluded that chronic MEL treatment reverses the induction effect of ISO on the mPTP, which was confirmed in experiments on mitochondrial swelling. The half-life of mitochondrial swelling in rats treated with MEL increased by 90%. ISO decreased this period by 34%, while the time required for RHM swelling under the action of ISO + MEL increased by 2 times.

Acuna-Castroviejo and colleagues showed that MEL improved the activity of the electron transport chain by decreasing the formation of ROS in complexes I and IV [123]. We identified changes in the levels of electron transport chain enzymes that may reflect changes in their activity in RHM isolated from each group of rats. We showed that MEL treatment reduced the level of the α subunit of complex V (ATPSA CV) by 11.6% (RHM from group 2 versus group 1), while ISO reduced its level by 30% (RHM from group 3 by compared with group 1). Pretreatment with MEL reduced the effect of ISO (by 23% compared with group 1). MEL increased the basic protein 2 level of complex III by 25%, while treatment with ISO alone and in combination with MEL increased the basic protein 2 level by 35% and 18%, respectively. MEL treatment reduced the NDUFB8 CI subunit level by 80%, and in combination with ISO, it was reduced by 14%. We did not notice any changes in the levels of the CIV and CII subunits in mitochondria isolated from groups 2, 3, and 4 compared to those from the control group.

We further evaluated ROS production in mitochondria isolated from control animals and animals treated with MEL and ISO. The mitochondria of the control rats as well as the mitochondria of the rats treated with MEL and ISO produced H_2_O_2_ at rates of ~50, 35, and 62 pmol/min/mg protein, respectively; however, the production of H_2_O_2_ in mitochondria from animals treated with MEL + ISO did not differ from the control. Ca^2+^ is known to accelerate the production of ROS in mitochondria through the activation of matrix NAD(P)-dependent dehydrogenases and the opening of the mPTP. We showed that, in the mitochondria of animals treated with ISO, the formation of H_2_O_2_ after the addition of Ca^2+^ was ~15% higher than in the control, and treatment with MEL did not decrease it. After 45 min of incubation, H_2_O_2_ production in the mitochondria of rats treated with ISO and MEL was 30% higher and 2.5 times lower than in the control, respectively, which was apparently associated with the acceleration and inhibition of mPTP opening, respectively. MEL reduced the production of H_2_O_2_ in the mitochondria of ISO-treated rats to a control level. Menadione-induced H_2_O_2_ production (~1000 pmol/min-1/mg protein) was reduced in the mitochondria of rats from groups 2 and 3 by 40% and 20%, respectively, and the effects of MEL and ISO were not additive. In contrast, antimycin A-dependent H_2_O_2_ production in complex III did not depend on MEL but ISO inhibited it by 20–30%. The release of superoxide anions from intact heart mitochondria was also suppressed in rats treated with ISO, and treatment with MEL did not restore this ability. These data suggest that both MEL and ISO are able to affect respiratory complex I, while only the ISO can affect complex III. In addition, MEL and ISO treatments produce opposite effects on mPTP opening and hence on ROS production.

Thus, we have shown that MEL prevents mitochondrial dysfunction associated with ISO-induced acute heart failure due to mPTP inhibition. The role of oxidation of substrates that supply electrons to the regions of the respiratory chain that follow complex I deserves further investigation.

The results of this study are consistent with previous data indicating that long-term administration of MEL in animals inhibits the opening of the mPTP in rat heart mitochondria [55]. In our experiments, ISO induced the opening of the mPTP in the RHM and, therefore, decreased the Ca^2+^ capacity of mitochondria by 40%, while MEL prevented the effect of ISO and increased the ability of mitochondria to retain Ca^2+^. MEL also demonstrated a protective effect in cardiac mitochondria isolated from ISO-treated rats.

In our studies, we showed that the activation of the mPTP in the mitochondria of the hearts of old animals is induced by lower threshold calcium concentrations. At the same time, in the mitochondria of old animals, the probability of mPTP transitioning to a state of low conductivity, leading to spontaneous release of superoxide anions from mitochondria, is significantly higher than in young animals. This occurs against the background of morphological changes in the mitochondria (a decrease in the number of cristae), indicating the suppression of mitochondrial functions. All of the above may indicate that, in the mitochondria of old rats, mPTP is in a pre-activated state and thus plays the role of a safety valve, preventing irreversible damage caused by Ca^2+^ and ROS.

MEL administration not only improves the condition of the heart muscle tissue of old animals but also prevents the onset of characteristic signs of ISO-induced heart failure, such as muscle fiber swelling, vacuolation of the sarcoplasm, granular degeneration, and the appearance of myolytic foci. Heart mitochondria isolated from old rats treated with MEL showed greater resistance to the opening of mPTP. Namely, the Ca^2+^ capacity of mitochondria increased and the rate of their swelling decreased. It also appears that pretreatment of animals with MEL partially inhibited the inductive effect of ISO on the mPTP. MEL partially prevented ISO-induced changes at the level of subunits of respiratory complexes III and V and sharply decreased the expression of the NDUFB8 subunit of complex I both in control mitochondria and in mitochondria from rats with heart failure, which led to inhibition of the production of ROS. Thus, our data show that melatonin has therapeutic potential for cardiovascular diseases.

## 4. Conclusions

**Figure 1:**
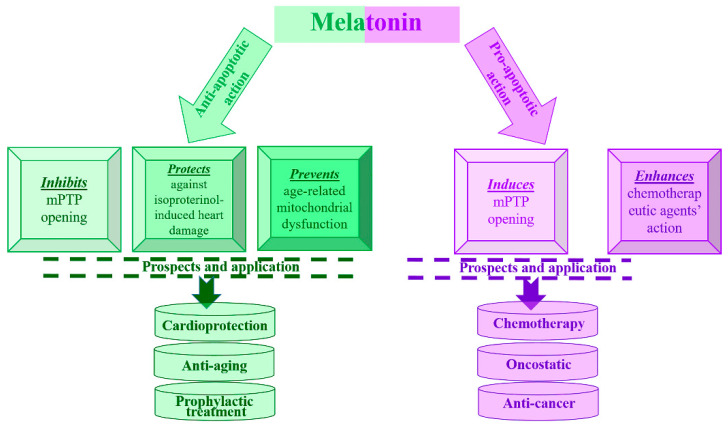
Scheme of the discovered opposite effects of melatonin and potential areas of its application.

Melatonin is capable of exerting both receptor-dependent and receptor-independent effects. It binds to known membrane receptors (MT1 and MT2) and, through various signaling pathways, can have various physiological effects. Melatonin receptors are capable of homo- and/or heterodimerization and interact with nuclear receptors (binding sites) [8]. Receptor-independent effects of MEL are mediated by the ability of MEL and its metabolites to scavenge ROS and reactive nitrogen species (RNS). These effects allow melatonin to protect against a wide range of toxins and processes that generate highly toxic reagents. Any cell can simultaneously respond to melatonin with both its receptor-mediated and receptor-independent actions. Why melatonin exhibits proapoptotic properties in cancer cells and anti-apoptotic properties in normal cells should be clarified in further studies. However, all of the above information indicates the potential of MEL to act as a pro- and antiapoptotic agent, which may have important biological, therapeutic, and pharmacological significance (Figure 1). MEL could be a very effective means both for prophylaxis, and its combined action with chemotherapeutic drugs already used in medicine could be used for cancer patients. The results of our research on the effect of MEL on mitochondrial functional status provide a theoretical basis for the development of future mitochondrial targeting drugs for the prevention and treatment of heart failure. Based on our research, recommendations can be developed for gerontologists regarding diet correction for the elderly. With age, the synthesis of intrinsic MEL decreases; therefore, it is necessary to increase the consumption of products containing tryptophan (the precursor of MEL).

## Data Availability

No new data were created or analyzed in this study. Data sharing is not applicable to this article.

## Abbreviations

ABT-737	navitoclax
ATP	adenosine triphosphate
ATRA	retinoic acid
APL	acute promyelocytic leukemia
ATPSA	ATP synthase F1 alpha subunit
CsA	cyclosporine A
Cyt c	cytochrome c
CYT	cytarabine
cAMP	cyclic adenosine monophosphate
CNPase	2’,3’-cyclic nucleotide 3’-phosphodiesterase
Cyp-D	Cyclophilin D
ETC	electron transport chain
ISO	isoprenaline hydrochloride
LV	left ventricle
MEL	melatonin
mPTP	mitochondrial permeability transition pore
NADH	nicotinamide-adenine-dinukleotidehidride
NDUFB8	NADH dehydrogenase [ubiquinone] 1 beta subcomplex subunit 8
RHM	rat heart mitochondria
RNS	reactive nitrogen species
ROS	reactive oxygen species
TSPO	translocator protein
VDAC	voltage-dependent anion channel
